# Recovery of Bioactive Compounds from Juçara Palm (*Euterpe edulis* Mart.) Fruit Residues Using Deep Eutectic and Conventional Solvents

**DOI:** 10.3390/plants14233693

**Published:** 2025-12-04

**Authors:** Ana Paula Stafussa, Jean Halison de Oliveira, Eduardo Cesar Meurer, Monica Regina da Silva Scapim, Grasiele Scaramal Madrona

**Affiliations:** 1 Postgraduate Program in Food Science, State University of Maringá (UEM), Maringá 87020-900, PR, Brazil; 2 Department of Food Engineering, State University of Maringá (UEM), Maringá 87020-900, PR, Brazil; jprsgrss@yahoo.com.br (M.R.d.S.S.); gsmadrona@uem.br (G.S.M.); 3 Department of Chemistry, State University of Maringá (UEM), Maringá 87020-900, PR, Brazil; jholiveira2@uem.br; 4 Department of Chemistry, Federal University of Paraná (UFPR), Jandaia Do Sul 86900-000, PR, Brazil; eduardo.meurer@ufpr.br

**Keywords:** green extraction, ultrasound, phenolic compounds, anthocyanins, antioxidant activity, ESI–MS/MS

## Abstract

This study aimed to develop an efficient and environmentally sustainable method for extracting bioactive compounds from juçara palm (*Euterpe edulis* Mart.) fruit residues using deep eutectic solvents (DES) and conventional solvents, combined with ultrasound-assisted extraction (UAE). Seven DES formulations based on choline chloride (ChCl) and different hydrogen bond donors (glycerol, glucose, and organic acids) were prepared, and their performance was compared with water, ethanol, and ethanol/water mixtures. The phenolic composition, anthocyanins and antioxidant activity of the extracts were determined using spectrophotometric assays (Folin–Ciocalteu, DPPH, ABTS, and FRAP) and ESI–MS/MS analysis. The results showed that DES exhibited higher efficiency in recovering total phenolic compounds, anthocyanins and ABTS compared to conventional solvents, particularly in the ChCl–glycerol system. ESI–MS/MS analyses monitored around 40 phenolic compounds, including phenolic acids, flavanones, flavonoids, and anthocyanins. Acidic solvents favored anthocyanin extraction and stability, while ethanol- and glycerol-based systems provided broader compound profiles. The use of DES proved to be a green and selective alternative for obtaining extracts rich in bioactive compounds, enhancing the value of juçara residues and contributing to the sustainability of the species production chain.

## 1. Introduction

In the Atlantic Forest region, several natural matrices exhibit high nutraceutical potential, among which is *Euterpe edulis* Mart., commonly known as juçara palm fruit. The valorization of juçara fruit and its by-products contributes to environmental sustainability and the conservation of Atlantic Forest biodiversity, as fruit harvesting does not require cutting the tree, whereas palm heart extraction threatens the species with extinction [1].

The phytochemical composition of juçara fruit is characterized mainly by flavonoids, especially anthocyanins, as well as phenolic acids. These compounds are known for their antioxidant activity and potential health benefits, including antidiabetic, antihyperlipidemic, and antimicrobial effects [2,3]. However, phenolic compounds such as anthocyanins are easily degraded and bioconverted into phenolic acids due to temperature, light, solvents, and pH changes, which affect their bioavailability [4]. To overcome these limitations, new extraction techniques and methodologies have been investigated, with deep eutectic solvents emerging as a promising alternative for obtaining bioactive extracts and for developing new green routes.

Deep eutectic solvents (DES) have been widely studied as sustainable alternatives for extracting bioactive compounds from plant matrices. These solvents stand out for reducing waste generation, replacing or minimizing the use of toxic reagents and solvents, and enabling the production of safe and high-quality extracts [5,6]. In this context, DES are regarded as efficient, eco-friendly, and alternative chemical systems.

The efficiency of the extraction process depends not only on the solvent properties but also on the extraction conditions. In this regard, ultrasound-assisted extraction (UAE) has gained attention as a promising technique to optimize the recovery of phenolic compounds, as it allows shorter processing times and reduced solvent consumption [7]. Moreover, UAE is considered a sustainable method due to its lower energy demand and reduced environmental impact [8].

Therefore, this paper aimed to develop an efficient and environmentally friendly method using deep eutectic solvents (DES) and conventional solvents, combined with ultrasonic technology, to extract bioactive compounds from juçara palm fruit residues, providing alternative routes for obtaining extracts rich in bioactive compounds.

## 2. Results and Discussion

### 2.1. Bioactive Compounds in Juçara Palm Fruit Residue Extracts

After preparing the different DES, solid–liquid extraction was carried out using an ultrasonic bath, as described in Section 3.4. The purpose of this assay was to maximize the recovery of bioactive compounds of interest from juçara palm fruit residues. For comparison, extractions were also performed using conventional solvents (ethanol, water, and ethanol/water (50/50, *v/v*)), along with the seven deep eutectic solvents (DES). The DES employed were generated using different hydrogen bond donors (HBDs), which provided each system with specific physicochemical characteristics that influence extraction performance. Table 1 presents the results for total phenolic compounds, anthocyanins, and antioxidant capacity determined by DPPH, ABTS, and FRAP assays.

The main limitation associated with deep eutectic solvents (DES), compared with conventional solvents, is their high viscosity, which can reduce mass transfer efficiency [9]. In this study, 20% (*w*/*w*) of water was added to each DES to lower viscosity while maintaining or even enhancing extraction efficiency for bioactive compounds. The addition of small amounts of water also shortens the time and temperature required for solvent preparation [10]. Furthermore, the presence of water alters DES polarity, increasing affinity for polar compounds and reducing solubility and diffusivity limitations through the solid matrix pores, thereby improving mass transfer and extraction performance [11].

The solvents used showed significant variation in antioxidant activity values across the assays for total phenolics (TPC), anthocyanins, ABTS•+, FRAP, and DPPH•. Overall, the eutectic solvents exhibited higher extraction efficiency for bioactive compounds, particularly in the ABTS•+, TPC, and anthocyanin assays, yielding significantly higher values (*p* < 0.05). Water also performed well in the DPPH• and FRAP assays, as did the ethanol–water mixture for FRAP.

Phenolic compounds were significantly better extracted using DES (Table 1), demonstrating the effectiveness of these solvents as alternatives for recovering antioxidant compounds. The stability of phenolics in DES may be attributed to intermolecular interactions, mainly hydrogen bonding between acidic phenolics in juçara extracts and the solvent constituents. These interactions enhance oxidative stability by restricting solute mobility and reducing oxygen exposure at the solvent–air interface [12].

The superior performance of DES in extracting phenolic compounds (Table 1) reflects their strong capacity to establish hydrogen-bond interactions with phenolic structures, which contributes to both efficient solubilization and enhanced oxidative stability [13]. According to Kim et al. [14], a choline chloride–glycerol (1:2, *w*/*w*) mixture exhibits solvatochromic parameters, including the $E_{T}^{N}$ index (Dimroth model) and Kamlet–Taft parameters, which describe polarity and polarizability (π*), hydrogen-bond donating (α), and accepting ability (β). These values are comparable to those of conventional solvents like water, suggesting that ChCl–glycerol DES possesses high polarity and a strong ability to form hydrogen bonds with the natural product matrix [13,14]. This explains the high extraction values for total phenolics, anthocyanins, and antioxidant activity observed for juçara residues with ChCl–glycerol DES.

Regarding DES formulated with ChCl and organic acids, their extraction efficiency for bioactive compounds varies according to the structural characteristics of the acids used, which impart distinct physicochemical properties to the resulting systems [11,15]. The use of different hydrogen bond donors (HBDs) modifies parameters such as viscosity, density, polarity, surface tension, and pH, all of which directly affect solvent performance and, consequently, extraction efficiency [16].

### 2.2. Phenolic Profile by MS/MS Evaluation

Characterization of the phenolic profile of juçara (*Euterpe edulis*) has garnered growing interest due to the nutritional and functional relevance attributed to these compounds. Studies on related species, such as açaí, have already demonstrated a richness in anthocyanins, flavonoids, and phenolic acids, while also revealing substantial variation as a function of the extraction methodology employed [17,18]. In this context, the present study evaluated the influence of ten distinct solvents on the phenolic composition of juçara, using targeted ESI-MS/MS in multiple reaction monitoring (MRM) mode as the analytical differential, a technique widely recognized for its high selectivity and sensitivity [19].

Approximately 40 phenolics belonging to different chemical classes were monitored, including benzoic and cinnamic acids, flavanones, flavonols, glycosylated derivatives, and anthocyanins (Table S1). However, only a subset of these compounds was effectively detected, reflecting the selectivity of extraction and the direct interplay between the plant matrix and the physicochemical properties of each solvent. This contrast between phenolics monitored and phenolics detected constitutes the starting point for the critical analysis developed herein. The solvents evaluated comprised five organic acids (malic, lactic, citric, tartaric, and acetic), two alternative polar media (glucose and glycerol), as well as water, ethanol/water, and neat ethanol. This panel was chosen to span a wide range of polarity, proticity, and hydrogen-bonding capacity, factors recognized as determinants of the extraction efficiency of phenolic compounds [20,21].

#### 2.2.1. Acid Solvents

Extracts obtained with acidic solvents displayed profiles characterized by a predominance of simple phenolic acids and the selective detection of anthocyanins. As shown in Table 2, malic and lactic acids were the only media capable of recovering cyanidin-3-O-arabinoside, an important marker of the anthocyanin class. This result is consistent with the stabilizing effect of low pH on the flavylium cation, recognized as the most stable structural form of anthocyanins in acidic environments [22,23]. This behavior arises from the acid–base equilibrium of anthocyanins: at low pH, the flavylium cation predominates, a structurally more stable and intensely colored species, which favors its detection only in strongly acidic media. The absence of this molecule in the other solvents confirms the dependence on acidity for its preservation during extraction.

Among the organic acids, tartaric acid stood out for trans-cinnamic acid. For p-hydroxybenzoic acid, the highest relative signal (peak area) occurred in ethanol, whereas tartaric and citric acids gave intermediate levels. In tartaric acid, vanillic acid also showed a notable signal, suggesting this medium can favor the release of phenylpropanoid-derived phenolics. In general, acids with greater capacity to form hydrogen bonds and to promote mild matrix hydrolysis tend to facilitate the release of simple phenolics [24].

Acetic acid exhibited a more restricted, yet still relevant, profile, with the presence of catechin, hesperetin, and p-hydroxybenzoic acid. The recovery of these compounds at moderate concentrations suggests that weaker acids may also be employed in selective strategies, provided they are applied under suitable conditions. Previous studies have indicated that acidic media promote partial hydrolysis of bound phenolics, contributing to their detection even in complex matrices [25].

Overall, the results obtained with acidic solvents highlight their importance both for the preservation of anthocyanins, whose stability depends on low pH, and for the efficient extraction of simple phenolic acids. These findings are consistent with previous reports in phenolic-rich plant matrices, in which acidic solvents were identified as strategic for broadening the chemical diversity extracted [26,27].

#### 2.2.2. Alcohol-Based, Aqueous, and Alternative Polar Solvents

Alcohol-based solvents exhibited the broadest performance among all those evaluated. Pure ethanol recovered the largest number of compounds, including phenolic acids (gallic, caffeic, p-hydroxybenzoic, trans-cinnamic), catechin, the flavonol quercetin, the flavanone hesperetin, and hesperetin-O-rutinoside. Naringenin, luteolin, and kaempferol were not detected in ethanol under our conditions. Ethanol’s breadth reflects its amphiphilic character: the hydroxyl group enables hydrogen-bonding with polar moieties, while the ethyl chain interacts with hydrophobic aromatic rings common in phenolics. By contrast, water yielded the highest signals for both the aglycone hesperetin and hesperetin-O-rutinoside (≈141.64 and 126.20 a.u., respectively), while ethanol and etha-nol/water provided lower yet relatively high signals (hesperetin ≈ 92.70 and 89.07 a.u.; hesperetin-O-rutinoside ≈ 95.14 and 90.18 a.u.). These trends are consistent with polarity-driven partitioning effects [19,28].

The ethanol/water mixture, although less diverse, displayed marked selectivity. Mean values indicated particularly high concentrations of hesperetin and hesperetin-O-rutinoside, even though other compounds were absent or at low levels. This behavior is related to the intermediate polarity of the mixture, which creates a favorable environment for specific flavanones but is less efficient for more hydrophobic compounds [19].

The aqueous extract, traditionally considered limited, yielded a singular result: hesperetin reached its highest relative concentration in this solvent (ca. 141 a.u.), nearly twice that observed in ethanol (ca. 93 a.u.) and higher than in all other solvents tested. This finding is relevant because it demonstrates that, even as a simple and low-cost solvent, water can be highly efficient for specific compounds. This efficiency may be attributed to the high solubility of molecules with free hydroxyl groups in hydrophilic media, combined with the low viscosity of the solvent, which facilitates diffusion and recovery of more polar phenolics.

Alternative solvents based on glucose and glycerol, in turn, revealed less broad but selective profiles. Glucose enabled the detection of p-hydroxybenzoic acid and trans-cinnamic acid, while glycerol was noteworthy for the recovery of caffeic acid, chlorogenic acid, hesperetin, and hesperetin-O-rutinoside at moderate concentrations. The high density of hydrogen-bonding sites in these solvents explains their preference for lower molecular weight compounds with multiple hydroxyl groups [29]. Although the diversity is reduced, these extracts show potential for applications in which selectivity is desirable, in addition to representing environmentally safer alternatives. Such selectivity is consistent with previous reports describing the distinctive stability and composition of phenolics in *Euterpe* species [30] and the variability observed in açaí by-product extracts [31].

Overall, the analysis of these solvents reveals two distinct patterns: broad diversity obtained with ethanol, and selectivity observed with ethanol/water, water, glucose, and glycerol. This complementarity highlights that solvent choice can be guided not only by chemical coverage but also by the technological purpose of the extract, whether for exploratory phytochemical studies or for targeted formulations [31].

#### 2.2.3. Complementary Chemical Analysis

The analysis by chemical classes revealed consistent patterns across the different solvents, allowing a better understanding of the selectivity observed in the extraction process. Simple phenolic acids were among the most recurrent classes. Trans-cinnamic acid and p-hydroxybenzoic acid stood out for their broad detection, particularly in acidic solvents such as citric and tartaric acids. Gallic and caffeic acids, on the other hand, were better recovered in alternative polar solvents, such as glycerol and in ethanol, suggesting a greater affinity with media capable of forming multiple hydrogen bonds. Chlorogenic acid, in contrast, appeared mainly in alcoholic solvents, in agreement with previous reports on its preferential extraction in hydroalcoholic systems [32,33].

Among the flavanones and flavonols, hesperetin was the most prominent compound, appearing in almost all solvents, but with maximum concentration in the aqueous extract. This result reinforces that compounds with polar functional groups may show high solubility in water, even though other flavonoids are not favored in this medium. Quercetin, luteolin, and kaempferol were better detected in ethanol, confirming the role of this solvent as a comprehensive extractor. Catechin, in turn, showed higher intensity in ethanol and acidic solvents, reflecting its dependence on environments capable of stabilizing multiple hydroxyl groups [33].

Glycosides were best represented in ethanolic extracts. The presence of hesperetin-O-rutinoside, luteolin-7-O-rutinoside, and kaempferol-hexosides in this solvent indicates that hydroalcoholic conditions favor the stability of these conjugated forms, possibly due to the combined effects of solubility and protection against degradation. Ethanol/water, in particular, selectively enhanced hesperetin-O-rutinoside, reaching higher values than in other solvents.

Anthocyanins exhibited a restricted pattern, being detected only in acidic solvents, especially in malic and lactic acid extracts. The presence of cyanidin-3-O-arabinoside in these media is consistent with the structural stability of anthocyanins in low-pH environments, where the flavylium cation predominates [34]. The absence of these molecules in ethanol and water confirms their high sensitivity to pH and solvent conditions.

Thus, the class-based analysis shows that while some simple phenolics and flavonoid aglycones are relatively ubiquitous, more labile compounds such as anthocyanins, or more polar ones such as glycosides, require specific extraction conditions. This pattern reinforces that the final extract composition results from a balance between solubility, stability, and solvent selectivity [35].

The comparison among the ten solvents evaluated shows that the final composition of juçara extracts is strongly modulated by the physicochemical characteristics of the extracting medium. Pure ethanol presented the most comprehensive profile, allowing the simultaneous detection of simple phenolic acids, flavanones, flavonols, and glycosides at relevant concentrations. This result confirms the role of ethanol as a reference solvent for the extraction of phenolics in complex plant matrices, due to its amphiphilic nature and safety for food and pharmaceutical applications [36].

The ethanol/water mixture exhibited lower diversity but notable selectivity, particularly for hesperetin-O-rutinoside, which reached high values in this system. Water, surprisingly, proved efficient for hesperetin, recording its highest relative concentration. This finding demonstrates that simple and low-cost solvents can present advantages for specific compounds, especially when selectivity is desired over diversity.

Acidic solvents confirmed their fundamental role in the preservation of anthocyanins, which were only detected in malic and lactic acid media. Moreover, they were effective for the extraction of simple phenolic acids such as trans-cinnamic and p-hydroxybenzoic acids, reinforcing the importance of pH in the release and stability of these molecules. Polyols and sugars, although less comprehensive, showed interesting selective profiles for low-molecular-weight compounds rich in hydroxyl groups, such as catechin. Another relevant point was the absence of higher-molecular-weight compounds, such as salvianolic acids, which were monitored but not detected in any solvent. This result indicates that such phenolics are not characteristic of juçara or are present at concentrations below the detection limit. This finding differentiates the chemical profile of this species from other plant matrices rich in high-molecular-weight compounds [29].

This study not only confirmed compounds already described for the *Euterpe* genus but also provided an unprecedented comparative analysis of the selectivity of different solvents for juçara phenolics. By combining coverage and selectivity data, this work makes an original contribution to the understanding of selective phenolic extraction, with implications both for the valorization of the species and for the development of strategies to support the industrial utilization of its extracts.

## 3. Materials and Methods

### 3.1. Chemicals and Reagents

All solutions were prepared using analytical-grade reagents and Milli-Q water. Antioxidant assay reagents (Trolox ≥ 97%, ABTS ≥ 98%, TPTZ ≥ 98%, DPPH ≥ 95%, Folin–Ciocalteu reagent) were obtained from Sigma-Aldrich Chemical Co. (St. Louis, MO, USA). Choline chloride (ChCl ≥ 99.26%,), glycerol (GL ≥ 99.5%), acetic acid (AA ≥ 99.7%), lactic acid (LA ≥ 85%), malic acid (MA ≥ 99%), citric acid (CA ≥ 99%), tartaric acid (TA ≥ 99%), glucose (GE ≥ 99%), and ethanol (≥99.8%) were purchased from Dinâmica Química Contemporânea Ltd. (Indaiatuba, SP, Brazil). All reagents/solvents were of analytical grade.

### 3.2. Raw Material

Fresh juçara palm fruits (*Euterpe edulis* Mart.) were obtained from a single batch provided by a local farmer in Maringá, Paraná, Brazil. Fully ripe (purple) fruits were selected, washed with potable water, and sanitized by immersion in sodium hypochlorite solution (200 mg L^−1^) for 15 min, followed by rinsing with running water. The fruits were manually macerated to separate the pulp, peel, and seed. The fruits were manually macerated to separate the pulp, peel, and seed. The pulp fraction was subsequently sieved through an 8-mesh sieve, and the material retained on the sieve (peel and seed) was designated as the residue. The seeds were discarded, and the peels were freeze-dried and portioned (250 g per package) and stored in plastic bags protected from light at −18 °C for later analyses.

### 3.3. Preparation of Deep Eutectic Solvents

Seven DES formulations were selected for extraction: ChCl:GL, ChCl:AA, ChCl:LA, ChCl:MA, ChCl:TA, ChCl:CA, and ChCl:GE. DES were prepared as described in the liter-ature [37,38,39], by mixing the hydrogen bond acceptor (HBA: ChCl) with the hydrogen bond donor (HBD: GL, AA, LA, MA, TA, CA, or GE) in a 1:2 molar ratio with 20% water. The mixtures were heated at 80 °C under stirring (Dubnoff bath, TE-053, Diadema, São Paulo, Brazil, 25 ± 5 rpm) until the formation of a transparent and homogeneous liquid (2–6 h).

### 3.4. Ultrasound-Assisted Extraction (UAE) of Bioactive Compounds

Extractions were carried out at a 1/20 (m/v) (peel/solvent) proportion, and performed in an ultrasonic bath (Ultronique, USC-1600 A, 120 W, 40 kHz, Indaiatuba, São Paulo, Brazil) with temperature control (50 °C) for 60 min. Solvents used were the prepared DES (Section 3.3), ethanol/water (50:50 *v*/*v*), and water. After extraction, the separation of the extracts from the plant matrix was carried out by vacuum-assisted filtration, which allowed efficient removal of solid residues.

### 3.5. Determination of Total Phenolic Content

Total phenolic content (TPC) was determined using the Folin–Ciocalteu colorimetric method [40], with modifications. A mixture of 0.125 mL of centrifuged extract and 0.125 mL of Folin–Ciocalteu reagent was allowed to react for 3 min before adding 2.25 mL of 20% sodium carbonate. Absorbance was measured at 765 nm (UV M51, BEL Engineering, Monza, MB, Italy). A calibration curve was prepared using gallic acid as the standard (y = 0.0055x + 0.06; R^2^ = 0.9957), and results were expressed as mg of gallic acid equivalents per 100 g of sample (mg GAE 100 g^−1^).

### 3.6. Determination of Monomeric Anthocyanins

Monomeric anthocyanin content (MAC) was determined by the pH differential method [41] using potassium chloride (KCl) and sodium acetate (C_2_H_3_NaO_2_). Absorbance was measured at 520 and 700 nm after 20 min incubation at 25 °C (Bel UV–Vis, model UV-M51, Piracicaba, São Paulo, Brazil). Results were expressed as mg of cyanidin-3-glucoside equivalents per 100 g of sample (mg cyanidin-3-glucoside 100 g^−1^), using Equation (1):

Anthocyanin pigment = (A × MW × Df × 103)/ε × λ(1)

where

A: (ABS 520 nm-ABS 700 nm) pH 1.0-(ABS 520 nm-ABS 700 nm) pH 4.5;
MW: 449.2 g mol^−1^ (cyanidin-3-glucoside molar weight);
Df: dilution factor;
103: conversion factor from g to mg;
ε: 26,900 L mol^−1^ cm^−1^ (cyanidin-3-glucoside molar extinction coefficient);
λ: 1 cm (cuvette path length).

### 3.7. In Vitro Antioxidant Activity

Antioxidant activity was evaluated using the DPPH (2,2-diphenyl-1-picrylhydrazyl) assay [42] at 515 nm (Bel UV–Vis, model UV-M51, Piracicaba, São Paulo, Brazil). A calibration curve was prepared using Trolox as the standard (y = 0.1101x + 3.8612; R^2^ = 0.9979), and results were expressed as µM of Trolox equivalents per gram of sample (µM TE g^−1^).

ABTS•+ radical scavenging activity was determined according to [43] using ABTS [2,2′-azino-bis(3-ethylbenzothiazoline-6-sulfonic acid)] and potassium persulfate (K_2_S_2_O_8_). Absorbance was measured at 734 nm after 6 min of incubation at 25 °C (Bel UV–Vis, model UV-M51, Piracicaba, São Paulo, Brazil). A calibration curve was prepared using Trolox as the standard (y = −0.0003x + 0.6889; R^2^ = 0.9925), and results were expressed as µM of Trolox equivalents per gram of sample (µM TE g^−1^).

The FRAP assay was performed following [44], using TPTZ (2,4,6-tris(2-piridil)-s-triazine), 0.3 M acetate buffer, and 20 mM ferric chloride. Absorbance was measured at 595 nm (Bel UV–Vis, model UV-M51, Piracicaba, São Paulo, Brazil). A calibration curve was prepared using ferrous sulfate as the standard (y = 0.0016x – 0.0368; R^2^ = 0.9901), and results were expressed as µM FeSO_4_ per gram of sample (µM FeSO_4_ g^−1^).

### 3.8. Characterization by Mass Spectrometry Using Electrospray (ESI-MS/MS)

Juçara fruit (*Euterpe edulis*) extracts were obtained using ten different solvent systems (see Section 3.4). For the preparation of samples, 0.01 g of each extract was accurately weighed and diluted to a final volume of 10 mL with HPLC-grade methanol (Merck, Darmstadt, Germany). The resulting solutions were homogenized by vortex mixing and, when necessary, further diluted in methanol to obtain suitable concentrations for analysis. All samples were filtered through a 0.45 μm Millex syringe filter (Millipore, Burlington, MA, USA) prior to injection into the chromatographic system.

Analyses were performed using a Quattro Premier XE Tandem Mass Spectrometer (Waters, Milford, MA, USA) equipped with an electrospray ionization (ESI) source. The extracts were introduced via direct inlet injection (without chromatographic separation) into the MS/MS system operating in multiple reaction monitoring (MRM) mode. Methanol containing 0.1% ammonium hydroxide (NH_4_OH) was used as the carrier solution at a flow rate of 200 μL·min^−1^, and the injection volume was 5 μL. The electrospray source was operated in the negative ionization mode [ESI (−)], with a capillary voltage of 2.0 kV. Source and desolvation temperatures were set at 110 °C and 200 °C, respectively. The cone voltage and collision energy were maintained at 20 V each. Argon was used as the collision gas at a pressure of 3.5 × 10^−3^ mbar.

The MRM mode was set to monitor precursor/product ion transitions specific to each phenolic compound targeted in this study. The complete list of monitored transitions, including parent and daughter ions, cone voltages, and collision energies, is provided in Supplementary Table S1. This protocol was adapted from Santos et al. [45], who successfully applied MRM-based MS/MS profiling for phenolic compounds in passion fruit (*Passiflora edulis*) seed extracts.

### 3.9. Statistical Analysis

All analyses were carried out in triplicate. Data were subjected to analysis of variance (ANOVA) and Tukey’s test (*p* < 0.05) using Sisvar 5.6 software. Calibration curves for antioxidant assays were generated using GraphPad Prism 5.

## 4. Conclusions

This study demonstrated that deep eutectic solvents (DESs) are a promising and environmentally sustainable alternative for extracting bioactive compounds from juçara palm fruit residues (*Euterpe edulis* Mart.). Among the systems tested, choline chloride–glycerol and choline chloride–glucose DESs exhibited the highest efficiency in recovering total phenolics and anthocyanins, along with strong antioxidant capacity. The addition of 20% water helped reduce viscosity and enhance mass transfer, improving extraction yield.

Phenolic profiling by MS/MS revealed that solvent choice strongly influences the selectivity and diversity of extracted compounds. Acidic solvents favored anthocyanin recovery and stability, while ethanol and glycerol enabled the extraction of a broader spectrum of phenolics, including phenolic acids, flavanones, and flavonols. These findings indicate that the rational selection of green solvents can be tailored to specific technological goals—whether to maximize chemical diversity or to target specific compounds.

Overall, the results highlight the potential of deep eutectic solvents as green tools for adding value to agro-industrial residues, fostering the development of sustainable processes and promoting the full utilization of *Euterpe edulis* biomass, a species of great ecological and economic relevance to the Atlantic Forest. Moreover, the optimized DES systems and their improved capacity to recover bioactive compounds provide a strong basis for potential applications in the food and pharmaceutical industries. In particular, they may facilitate the development of natural antioxidant ingredients, support the formulation of functional products, and contribute to the implementation of more environmentally sustainable extraction technologies. These considerations further reinforce the practical relevance of our findings and their potential translation into real-world applications.

## Figures and Tables

**Table 1:** Mean values for Total phenolic compounds, anthocyanins, and antioxidant capacity (DPPH, FRAP, and ABTS) of juçara palm fruit residue extracted with different solvents.

SAMPLE	TPC	TMA	DPPH	FRAP	ABTS
ChCl:GL	5372.99 ^a^ ± 610.58	174.00 ^a^ ± 3.53	75.25 ^b,c^ ± 0.60	17.90 ^a^ ± 0.04	22.68 ^a^ ± 1.44
ChCl: AA	5189.33 ^a^ ± 515.33	145.01 ^b^ ± 6.09	84.03 ^a^ ± 0.15	17.63 ^a^ ± 0.06	20.35 ^a^ ± 1.29
ChCl:LA	3394.32 ^b^ ± 290.97	121.67 ^c^ ± 5.12	44.79 ^e^ ± 3.04	13.62 ^d^ ± 0.43	22.66 ^a^ ± 1.12
ChCl:MA	5140.73 ^a^ ± 784.95	103.30 ^d^ ± 6.53	39.77 ^e,f^ ± 2.30	15.54 ^c^ ± 0.62	12.55 ^b^ ± 2.24
ChCl:CA	4315.86 ^a,b^ ± 956.94	53.09 ^e^ ± 7.46	38.65 ^e,f^ ± 5.10	13.80 ^d^ ± 0.27	5.03 ^c^ ± 0.59
ChCl:TA	4427.65 ^a,b^ ± 140.27	28.39 ^f^ ± 3.05	31.52 ^f^ ± 4.98	14.31 ^d^ ± 0.54	6.26 ^c^ ± 0.55
ChCl:GE	5690.52 ^a^ ± 557.97	127.17 ^b,c^ ± 3.96	72.64 ^c,d^ ± 4.69	16.54 ^b^ ± 0.15	1.13 ^d^ ± 0.20
Water	1718.85 ^c^ ± 53.31	49.32 ^e^ ± 3.05	83.17 ^ab^ ± 0.52	17.77 ^a^ ± 0.04	12.85 ^b^ ± 1.4
Ethanol	760.28 ^c^ ± 8.33	13.42 ^f^ ± 1.17	43.93 ^e^ ± 1.10	16.12 ^b,c^ ± 0.17	1.18 ^d^ ± 0.66
Ethanol/Water	1762.82 ^c^ ± 16.64	87.35 ^d^ ± 2.34	66.04 ^d^ ± 1.09	18.04 ^a^ ± 0.03	12.67 ^b^ ± 0.70
CV (%)	13.47	5.14	5.19	1.96	9.93
*p*-value	<0.01	<0.01	<0.01	<0.01	<0.01

Data are expressed as means ± standard deviation. Means with different letters in the same column are significantly different for p ≤ 0.05; Tukey’s test. CV (%) is the coefficient of variation. TPC (Total Phenolics Compounds) expressed in mg GAE/100 g); TMA (Total Monomeric Anthocyanins expressed in mg cyanidin-3-glucoside 100 g^−1^); DPPH expressed in % Efficiency of free radical scavenging; ABTS expressed in m mol TE 100 g^−1^ and FRAP expressed in m mol Fe_2_SO_4_ 100 g^−1^. ChCl:GL (Choline chloride–glycerol), ChCl:AA (Choline chloride–acetic acid), ChCl:LA (Choline chloride–lactic acid), ChCl:MA (Choline chloride–malic acid), ChCl:CA (Choline chloride–citric acid), ChCl:TA (Choline chloride–tartaric acid), ChCl:GE (Choline chloride–glucose).

**Table 2:** Phenolic compounds identified by MRM in juçara (*Euterpe edulis*) extracts: mean peak area (a.u.) ± SD, *n* = 3.

Compounds	[M−H]− (*m/z*)	Fragment (*m/z*)	1	2	3	4	5
trans-Cinnamic acid	147	103	47.54 ± 7.81	ND	ND	ND	72.26 ± 4.52
p-Hydroxybenzoic acid	137	93	33.24 ± 4.25	ND	22.36 ± 3.81	15.79 ± 2.96	30.17 ± 5.73
Catechin	289	245	42.89 ± 8.03	ND	ND	18.35 ± 2.91	ND
Cyanidin-3-O-arabinoside	418	287	64.31 ± 11.82	59.27 ± 8.90	ND	ND	ND
Hesperetin-O-rutinoside	609	301	82.07 ± 9.44	76.14 ± 10.81	60.17 ± 8.53	69.12 ± 7.46	88.23 ± 10.38
Hesperetin	301	151	78.14 ± 10.65	72.20 ± 9.54	55.28 ± 7.18	66.09 ± 8.01	84.31 ± 11.77
p-Coumaric acid	163	119	ND	23.47 ± 4.16	19.87 ± 3.91	ND	ND
Luteolin-7-O-glucuronide	461	285	ND	34.19 ± 6.72	29.41 ± 5.39	ND	26.28 ± 4.12
Naringenin	271	151	ND	ND	17.64 ± 3.55	ND	ND
Kaempferol-3-O-malonylhexoside	533	285	ND	ND	ND	21.48 ± 3.17	ND
Kaempferol	285	151	ND	ND	ND	ND	19.73 ± 2.88
Compounds	[M−H]− (*m/z*)	Fragment (*m/z*)	6	7	8	9	10
trans-Cinnamic acid	147	103	85.61 ± 13.61	33.04 ± 3.91	40.75 ± 8.12	21.90 ± 4.72	56.38 ± 9.43
p-Hydroxybenzoic acid	137	93	27.08 ± 4.17	ND	ND	28.11 ± 4.65	39.07 ± 7.91
Catechin	289	245	ND	ND	ND	ND	31.24 ± 5.08
Cyanidin-3-O-arabinoside	418	287	ND	ND	ND	ND	ND
Hesperetin-O-rutinoside	609	301	65.12 ± 8.89	47.90 ± 7.91	90.18 ± 28.16	126.20 ± 11.07	95.14 ± 12.83
Hesperetin	301	151	61.07 ± 9.42	44.19 ± 7.33	89.07 ± 13.36	141.64 ± 5.31	92.70 ± 10.42
p-Coumaric acid	163	119	ND	ND	ND	ND	ND
Luteolin-7-O-glucuronide	461	285	ND	ND	ND	ND	ND
Naringenin	271	151	ND	ND	ND	ND	ND
Kaempferol-3-O-malonylhexoside	533	285	ND	ND	ND	ND	ND
Kaempferol	285	151	ND	ND	ND	ND	ND
Vanillic acid	167	108	37.15 ± 8.36	ND	ND	ND	ND
Caffeic acid	179	135	ND	28.39 ± 4.92	ND	ND	33.08 ± 6.10
Chlorogenic acid	353	191	ND	24.91 ± 3.80	ND	ND	ND
Quercetin	301	151	ND	ND	ND	ND	29.71 ± 4.21
Gallic acid	169	125	ND	ND	ND	ND	22.44 ± 3.66

ND = not detected; *n* = 3; units expressed as peak area (a.u.). Solvent legend: 1 = Choline chloride–Malic acid; 2 = Choline chloride–Lactic acid; 3 = Choline chloride–Citric acid; 4 = Choline chloride–Acetic acid; 5 = Choline chloride–Glucose; 6 = Choline chloride–Tartaric acid; 7 = Choline chloride–Glycerol; 8 = Ethanol/Water; 9 = Water; 10 = Ethanol.

## Data Availability

The datasets supporting the conclusions of this article are included within the manuscript.

## Supplementary Materials

The following supporting information can be downloaded at https://www.mdpi.com/article/10.3390/plants14233693/s1. Table S1: Multiple Reaction Monitoring (MRM) transitions of phenolic compounds monitored in ESI–mode.
