# Supplementary material for: Recovery of Bioactive Compounds from Juçara Palm (Euterpe edulis Mart.) Fruit Residues Using Deep Eutectic and Conventional Solvents
Source: Plants (Basel). 2025 Dec 4;14(23):3693. doi: 10.3390/plants14233693 (PMC12693893; doi:10.3390/plants14233693)
Supplement: Supplementary file 1 [file plants-14-03693-s001.zip › plants-3978255-supplementary.pdf]

SUPPLEMENTARY MATERIAL

The table includes all targeted phenolic standards screened in this study, with their respective parent and daughter ions, cone voltage, and collision energy parameters.

Table S1. Multiple Reaction Monitoring (MRM) transitions of phenolic compounds monitored in ESI–mode.

**Compounds**	**Parent (m/z)**	**Daughter (m/z)**	**Cone (V)**	**Collision (V)**
p-Hydroxybenzoic acid	137	93	20	20
trans-Cinnamic acid	147	103	20	20
Protocatechuic acid	153	109	20	20
p-Coumaric acid	163	119	20	20
Vanillic acid	167	108	20	20
Gallic acid	169	125	20	20
Caffeic acid	179	135	20	20
Ferulic acid	193	134	20	20
Syringic acid	197	182	20	20
Chlorogenic acid	353.4	135	20	20
Chlorogenic acid*	353.4	191	20	20
3-O and 5-O-Caffeoylquinic acid*	353.2	191	20	20
Rosmarinic acid	359	161	20	20
Apigenin	269	107	20	20
Kaempferol	285	151	20	20
Kaempferol	285	145	20	20
Catechin	289	245	20	20
Isorhamnetin	315	300	20	20

Myricetin	317	151	20	20
Luteolin	285	151	20	20
Luteolin	285	259	20	20
Hesperetin	301	151	20	20
Quercetin	301	151	20	20
Quercetin	301	179	20	20
Quercetin	301	269	20	20
Quercetin	301	286	20	20
Naringenin	271	151	20	20
Quercetin-3-O-rutinoside	609	300	20	20
Hesperetin-O-rutinoside	609	301	20	20
Luteolin-7-O-glucuronide	461	285	20	20
Luteolin-7-O-rutinoside	593	285	20	20
Luteolin-O-diglucuronide	637	285	20	20
Kaempferol-hexoside	447	285	20	20
Kaempferol-3-malonyl-hexoside	533	285	20	20
Cyanidin-3-O-arabinoside	418	287	20	20
Cyanidin-3-O-glucoside	447	285	20	20
Cyanidin-3,5-O-diglucoside	610	287	20	20
Cyanidin-3,5-O-diglucoside	610	448	20	20
Naringenin-O-rutinoside	579	271	20	20
Protocatechuic acid glucoside	315	153	20	20

Eriodictyol-O-hexoside	449	287	20	20
Eriodictyol-O-rutinoside	595	287	20	20
Diosmin	607	299	20	20
Salvianolic acid A	493	295	20	20
Salvianolic acid B	717	393	20	20
Salvianolic acid D	717	399	20	20
Salvianolic acid E	717	537	20	20
Salvianolic acid H	537	339	20	20

**Compounds sharing identical precursor→product ion transitions cannot be distinguished under direct-infusion MS/MS.*
